# Efficient Privacy-Preserving *K*-Means Clustering from Secret-Sharing-Based Secure Three-Party Computation

**DOI:** 10.3390/e24081145

**Published:** 2022-08-18

**Authors:** Weiming Wei, Chunming Tang, Yucheng Chen

**Affiliations:** 1 School of Mathematics and Information Science, Guangzhou University, Guangzhou 510006, China; 2 School of Mathematics, Jiaying University, Meizhou 514015, China

**Keywords:** privacy-preserving *K*-means clustering, secure outsourced computation, replicated secret sharing, semi-honest model

## Abstract

Privacy-preserving machine learning has become an important study at present due to privacy policies. However, the efficiency gap between the plain-text algorithm and its privacy-preserving version still exists. In this paper, we focus on designing a novel secret-sharing-based *K*-means clustering algorithm. Particularly, we present an efficient privacy-preserving *K*-means clustering algorithm based on replicated secret sharing with honest-majority in the semi-honest model. More concretely, the clustering task is outsourced to three semi-honest computing servers. Theoretically, the proposed privacy-preserving scheme can be proven with full data privacy. Furthermore, the experimental results demonstrate that our proposed privacy version reaches the same accuracy as the plain-text one. Compared to the existing privacy-preserving scheme, our proposed protocol can achieve about 16.5×–25.2× faster computation and 63.8×–68.0× lower communication. Consequently, the proposed privacy-preserving scheme is suitable for secret-sharing-based secure outsourced computation.

## 1. Introduction

With the rapid development of machine learning, Machine Learning as a Service (MLaaS) has become a popular business. Nowadays, machine learning is also widely applied in different fields, such as finance, healthcare, image recognition, and so on. Major companies such as Microsoft, Google, Amazon, etc. are beginning to provide cloud-based MLaaS. In general, these services allow the machine learning algorithms to be updated and improved via input data from their users. In order to gain high-precision model, companies tend to come together and train a common model using their datasets.

However, with the improvement of awareness of privacy, the problems caused by data privacy leakage have become increasingly prominent. On the one hand, the user, who uses the MLaaS service, hopes that the service is conducted without revealing any information of their queries and prediction result. On the other hand, companies want to train a common model without sharing their dataset. Therefore, it is important to find a secure way for privacy-preserving machine learning (PPML) to proceed.

Privacy-preserving machine learning can be tracked back to privacy-preserving data mining, which was firstly introduced by Lindell and Pinkas [1]. Since then, more and more researchers have put focus on privacy-preserving machine learning.

The dataset can be divided into two main types: labeled data and unlabeled data. Generally speaking, the former usually uses supervised learning algorithms when training the model, while the latter unsupervised learning algorithm [2]. In recent years, most solutions of PPML only consider the supervised learning algorithm, and there is less consideration of the unsupervised learning algorithm.

As an unsupervised machine learning technique, similar input records are grouped into clusters while records belonging to different clusters should be maximally different [3]. In this work, we focus on clustering, which plays an extremely important role in data processing and analysis. The goal of clustering is to divide given unlabeled data into several disjoint subsets, such that each subset has similar properties to each other.

The *K*-means algorithm is one of the most well-known clustering algorithms. *A privacy-preserving K-means clustering, which has full data privacy, allows the parties to cluster their combined datasets without revealing any other information except for the final centroid* [3]. In other words, the information of intermediate centroids, cluster assignments, and cluster sizes should be protected in the protocol. Although there are several works for privacy-preserving *K*-means clustering at present, only few of them consider both full data privacy and efficiency. This raises the question:

*Could we find a way to achieve both full data privacy for security and efficiency for practicability?*

As we will show below, the answer is yes with our replicated secret-sharing-based *K*-means clustering protocol.

### 1.1. Related Work

In general, different algorithms have different privacy-preserving tools. For example, the chaotic system is a popular tool for image encryption [4]. Privacy-preserving *K*-means clustering falls into one of two categories: (i) homomorphic encryption-based and (ii) secure multiparty computation-based.

Homomorphic encryption (HE) was first proposed by Rivest et al. [5] in 1978. Homomorphic encryption is an encryption scheme where there exists a homomorphism relationship between operations on the plaintext and operations on the ciphertext, such that one can operation on the ciphertext can proceed without leaking any information of plaintext and it obtains the same effect as operation on plaintext after decrypting the result. HE is also widely applied in secure outsourced computation. HE can be divided into fully homomorphic encryption (FHE) and partially homomorphic encryption. FHE supports arbitrary computation on ciphertexts without any limitation. The first feasible FHE scheme was proposed by Gentry [6] in 2009, but it is inefficient. Instead of using inefficient FHE, many researchers adopt efficient partially homomorphic encryption, which only supports homomorphic addition or homomorphic multiplication. For example, RSA [5], Paillier [7], and ElGamal [8] are common partially homomorphic encryptions.

Generally speaking, *an HE-based scheme provides full data privacy, as long as the underlying HE cryptosystem is secure*. The first HE-based *K*-means clustering scheme was given by Vaidya and Clifton [9] in 2003, but it does not satisfy full data privacy. In 2007, Bunn and Ostrovsky [10] presented a two-party privacy-preserving *K*-means scheme based on additive homomorphic encryption that guarantees full data privacy in the semi-honest model. In 2015, Rao et al. [11] proposed a parallelable outsourced distributed clustering protocol based on Paillier homomorphic encryption in the federated cloud environment, but their work is inefficient due to its bit-array-based comparison; hence, Kim and Chang [12] improved it with a new secure comparison protocol. Jäschke and Armknecht [13] proposed *K*-means clustering based on FHE over the torus (TFHE) [14], which provides full privacy guarantees. Cai and Tang [15] proposed their *K*-means algorithm based on Liu’s homomorphic encryption [16], which has been proven insecure [17]. Unfortunately, all of these schemes do not scale for large datasets due to the heavily homomorphic operations.

Secure two-party computation (2PC) was first presented by Yao [18] in 1982 and extended to multiparty computation (MPC) by Goldreich et al. [19] in 1987. According to the parties number, we construct 2PC protocol with Yao’s garbled circuit (GC) [20], while MPC is done with secret sharing (SS). To further enhance efficiency, *t*-out-of-*n* threshold secret sharing has been applied in recent years. Moreover, the algebraic structure is another important optimized direction. For example, Shamir’s secret sharing (SSS) is a famous threshold secret sharing method [21], but it generally works for finite field, such as prime field $Z_{p}$, which is inefficient compared with several protocols operating over the-power-of-two ring $Z_{2^{\ell }}$ in PPML. This is because the latter takes full advantage of the underlying CPU architecture.

In this work, we only focus on SS-based *K*-means clustering. Doganay et al. [22] proposed distributed privacy preserving *K*-means clustering with additive secret sharing (ASS), but this work reveals the final cluster assignments to parties. Patel et al. [23,24] proposed their *K*-means algorithm under different security model. Upmanyu et al. [25] and Baby and Chandra [26] respectively presented a distributed threshold secret sharing scheme based on the Chinese remainder theorem (CRT-SS). However, none of them provide full privacy guarantees as shown in [3]. In 2020, Mohassel et al. [27] presented 2PC *K*-means clustering protocol with 2-out-of-2 additive secret sharing, and although it provides full data privacy, it is inefficient in terms of computation and communication overhead, because their work heavily relies on garbled circuit and oblivious transfer (OT). Therefore, this scheme is not practical for large-scale clustering tasks.

As shown above, most of the existing privacy-preserving *K*-means clustering protocols take no account of full data privacy. In addition, the gap of efficiency still exists compare with plaintext training. Algorithm inefficiency also limits its practicality, especially for large-scale training tasks. In this work, we want to construct privacy-preserving *K*-means clustering, which has full data privacy for security and high efficiency for practicality.

### 1.2. Our Contributions

In this work, we only focus on SS-based *K*-means clustering schemes. We provide the comparison with existing SS-based *K*-means clustering in Table 1. We propose an efficient three-party computation protocol for privacy-preserving *K*-means clustering. Concretely, our contributions are described as follows:

- Our protocol provides full privacy guarantees, which allows different computing parties to cluster the combined datasets without revealing any other information except the final centroids.
- Our protocol is based on replicated secret sharing (RSS), which is a 2-out-of-3 threshold secret sharing proposed by Araki et al. [28] and is suitable for constructing efficient protocol over $Z_{2^{\ell }}$. Our protocol is secure against a single corrupt server under a semi-honest model. We analyze the security with universal composition framework [29].
- The experimental results demonstrate that our protocol reaches the same accuracy as the plaintext *K*-means clustering algorithm. With the fast network, our privacy-preserving scheme can deal with datasets of million points in an acceptable time.

### 1.3. Roadmap

The remaining sections are organized as follows. In Section 2, we give the definition of basic notation, threat model, and security assumption of secure computation, and plaintext algorithm related to *K*-means clustering. In Section 3, we give the cryptographic building blocks. In Section 4, we propose our efficient three-party protocol construction. We give detailed security analysis of our protocol in Section 5. Then, we report the experimental results of our construction in Section 6. Finally, we conclude this paper in Section 7.

## 2. Preliminaries

### 2.1. Basic Notation

We denote the party *i* by $P_{i}$ for each i∈{1,2,3}. For simplicity, we define $P_{0}=P_{3}$, and $P_{4}=P_{1}$ in the context. $x\in_{R}F$ is chosen uniformly at random from finite set F. We write a bold letter v to denote a *d*-dimension vector. The *j*-th component of vector v is $v_{j}$. If *x* is a *ℓ*-bit number, then x[i] is its *i*-th bit. Let κ be the security parameter. We use [n] to denote set {1,⋯,n}. Furthermore, we assume all float-point data are encoded as *ℓ*-bit fixed-point number with *f*-bit precision, where f<ℓ.

### 2.2. Threat Model and Security Assumption

Our protocol follows a static and semi-honest model [30] under the honest-majority setting, i.e., the adversary A only corrupts a single and fixed party during protocol executing. In this setting, the corrupted party follows protocol honestly and wants to learn the input of other parties from received messages. Therefore, the semi-honest model is also called the passive model. Furthermore, we assume the parties communicate with other parties through a secure channel and the network is synchronized.

We prove security using a universally composable framework [29] in the ideal–real paradigm [30]. Let F be the ideal functionality executed by a trusted third party (TTP) in the ideal world, and ∏ be the real protocol executed by all parties in the real world. In the ideal world, there is a simulator Sim that plays as adversary A. Let *C* be the set of corrupted parties and $x_{i}$ be $P_{i}$’s input. We define the ideal interaction and the real interaction as follows:

- ${\mathrm{Ideal}}_{F,\mathrm{Sim}}\left(\kappa ,C;x_{1},\cdots ,x_{n}\right)$: Compute $\left(y_{1},\cdots ,y_{n}\right)\leftarrow F\left(x_{1},\cdots ,x_{n}\right)$;
  Output $\mathrm{Sim}\left(C,\left\{\left(x_{i},y_{i}\right),i\in C\right\}\right),\left(y_{1},\cdots ,y_{n}\right)$, where $y_{i}$ is $P_{i}$’s output.
- ${\mathrm{Real}}_{\prod ,A}\left(\kappa ,C;x_{1},\cdots ,x_{n}\right)$: Run the protocol ∏;
  Output $\left\{{\mathrm{View}}_{i},i\in C\right\},\left(y_{1},\cdots ,y_{n}\right)$, where ${\mathrm{View}}_{i}$ is the final view of $P_{i}$.

We say the protocol ∏ securely computes the functionality F in the semi-honest model, if the view of simulator in the ideal world is indistinguishable from the view of adversary in the real world. We refer the reader to [30] for more details.

### 2.3. The *K*-Means Clustering Algorithm

Given dataset $D=\{P_{1},\cdots ,P_{n}\}$ with *n* data points, each point $P_{i}$ is a *d*-dimension vector $\left(P_{i1},\cdots ,P_{id}\right)$. We form n×d matrix P. A standard *K*-means clustering algorithm includes the following steps [10,27]:

- Cluster centroids initialization: randomly choose *K* different points as initialized centroids $\phi_{1},\cdots ,\phi_{K}$ for *K* groups, where $\phi_{k}$ is *d*-dimension vector $\left(\phi_{k1},\cdots ,\phi_{kd}\right)$, k∈[K].
- Repeat the following until the stopping criterion (Lloyd’s steps):
  (a) For i∈[n],k∈[K], compute the Euclidean distance between point $P_{i}$ and centroids $\phi_{k}$ by
    (1) $$X_{ik}=\sqrt{\sum_{j=1}^{d}{\left(P_{ij}-\phi_{kj}\right)}^{2}}.$$
  (b) Assign each data point $P_{i}$ to the closest cluster $m_{i}$ for i∈[n]. This can be done by computing $k_{i}\leftarrow \arg\;\min\left\{X_{i1},\cdots ,X_{iK}\right\}$ firstly, and then generate a *K*-dimension one-hot vector $c_{i}$ where ‘1’ indicates the $k_{i}$-th component of vector $\left(X_{i1},\cdots ,X_{iK}\right)$. We form K×n matrix C such that the *i*-th column of C is the one-hot vector $c_{i}$. Let $m_{k}$ be the *k*-th row of C.
  (c) Recalculate the average of the points in each cluster. For each cluster k∈[K], compute new cluster center with
    (2) $$\varphi_{k}=\frac{m_{k}\cdot P}{D_{k}},$$
    where $D_{k}=\sum_{i=1}^{n}m_{ki}$ is the point number of *k*-th cluster.
  (d) Check the stopping criterion and update the new cluster center with the average. For each k∈[K], compute the Euclidean distance between $\varphi_{k}$ and $\phi_{k}$ at first, and then the squared error can be computed by
    (3) $$e=\sum_{k=1}^{K}e_{k}=\sum_{k=1}^{K}\sqrt{\sum_{j=1}^{d}{\left(\varphi_{kj}-\phi_{kj}\right)}^{2}}.$$
    Given a small error ε, if e≥ε, then update $\phi_{k}$ with $\varphi_{k}$. Otherwise, stop the criterion and output $\varphi_{k}$.

## 3. Building Blocks

This section gives the building blocks for our privacy-preserving *K*-means clustering protocol.

### 3.1. Correlated Randomness

In order to generate randomness among parties without any interaction, similar to [28,31,32], correlated randomness is introduced to this work.

Let $F:Z_{2}^{\kappa }\times Z_{2}^{\kappa }\to Z_{2^{\ell }}$ be a secure pseudo-random function (PRF). count is a counter maintained by the parties and updated after every PRF invocation. All parties run a one-time setup to establish PRF keys. The one-time setup can be done by letting each $P_{i}$ choose a random key $k_{i}$, and then sending it to $P_{i-1}$ for i∈[3]. Namely, each $P_{i}$ has the random PRF keys $k_{i}$ and $k_{i-1}$ after the setup. In this way, the parties can generate the following correlated randomness locally:

- *3-out-of-3 randomness*: $P_{i}$ holds $\alpha_{i}=F_{k_{i}}\left(\mathrm{count}\right)-F_{k_{i-1}}\left(\mathrm{count}\right)$.
- *2-out-of-3 randomness*: $P_{i}$ holds $\left(\alpha_{i},\alpha_{i-1}\right)=\left(F_{k_{i}}\left(\mathrm{count}\right),F_{k_{i-1}}\left(\mathrm{count}\right)\right)$.

Note that 3-out-of-3 randomness has the property that $\alpha_{1}+\alpha_{2}+\alpha_{3}\equiv 0\;\mathrm{mod}\;2^{\ell }$, which is known as *zero sharing*.

### 3.2. Replicated Secret Sharing

Replicated secret sharing (RSS) was first proposed by Ito et al. [33]. In CCS’16, Araki et al. [28] presented 2-out-of-3 replicated secret sharing scheme, which has high throughput and low latency. As a famous 3PC framework, ABY3 is also based on this variant over 2-power ring $Z_{2^{\ell }}$ [31]. Our protocol also builds on ABY3. Let *m* be a general modulus, and we describe this replicated secret sharing as follows.

- ⟦x⟧←share(x): To share a secret $x\in Z_{m}$, the dealer samples three random values $x_{1},x_{2},x_{3}\in_{R}Z_{m}$ under the constraint that $x\equiv x_{1}+x_{2}+x_{3}\;\mathrm{mod}\;m$. For i∈[3], $P_{i}$ gets $\left(x_{i},x_{i+1}\right)$. We write $⟦x⟧:=(x_{1},x_{2},x_{3})$.
- x←reconstruct(⟦x⟧,P): To reveal ⟦x⟧ to all parties, $P_{i}$ sends $x_{i}$ to $P_{i+1}$, then each party reconstructs *x* locally by computing $x_{1},x_{2},x_{3}\in_{R}Z_{m}$. To reveal ⟦x⟧ only to $P_{i}$, $P_{i-1}$ sends $x_{i-1}$ to $P_{i-1}$ which reconstructs *x* locally.

In this work, *m* can be a different modulus. When $m=2^{\ell }$, we call that *arithmetic share* and denote it as ⟦x⟧ or $⟦x{⟧}^{A}$. When m=2, we call that *boolean share* and denote it as $⟦x{⟧}^{B}$.

The linear operation between two shares can be computed locally because of the linearity property. This means that given public constant a,b,c and two shares $⟦x⟧=\left(x_{1},x_{2},x_{3}\right),⟦y⟧=\left(y_{1},y_{2},y_{3}\right)$, ⟦ax±by±c⟧ can be locally computed as $\left(ax_{1}\pm by_{1}\pm c,ax_{2}\pm by_{2},ax_{3}\pm by_{3}\right)$. In order to compute the shares of multiplication ⟦z⟧=⟦xy⟧, the parties generate zero sharing locally at first, and then $P_{i}$ locally computes 3-out-of-3 share $z_{i}=x_{i}y_{i}+x_{i+1}y_{i}+x_{i}y_{i+1}+\alpha_{i}$ for each i∈[3]. Finally, *resharing* is performed by the parties for 2-out-of-3 sharing semantics; this can be done by $P_{i}$ that sends $z_{i}$ to $P_{i+1}$. It is easy to see that each party only sends 1 ring element per multiplication. Compare that with ASS in the 3PC setting, wherein RSS reduces 50% communication overhead. In this context, we denote RSS multiplication protocol as $\prod_{\mathrm{Mul}}$.

If the dealer wants to share a random value $r\in_{R}Z_{2^{\ell }}$ for *j*-th time to all parties, the PRF key from zero sharing can be used. In particular, $P_{i}$ lets $r_{i}=F_{k_{i}}\left(j\right)$ and $r_{i-1}=F_{k_{i-1}}\left(j\right)$. If $P_{1}$ wants to share his private input *x* to all parties, the parties first generate another zero sharing $\left(\beta_{1},\beta_{2},\beta_{3}\right)$, then define the share of *r* as $⟦r⟧:=(\beta_{1}+x,\beta_{2},\beta_{3})$, and $P_{i}$ sends *x* to $P_{i-1}$ in the end.

Note that the appearance of decimals in the computation is unavoidable in the computation, while secret sharing only works on the integer field. To represent a real number $\bar{x}\in R$, we use a fixed-point representation with *f*-bit precision [34]. We scale $\bar{x}$ by a factor of $2^{f}$ and represent the rounded integer $x=\lfloor 2^{f}\cdot \bar{x}\rfloor$ as a *ℓ*-bit signed integer over $Z_{2^{\ell }}$. However, the multiplication result $⟦z^{\prime }⟧$ between two shares ⟦x⟧ and ⟦y⟧ will have 2f-bit precision.

To reduce precision from 2f to *f*, Mohassel and Rindal [31] introduce two probability truncation techniques to truncate the *f*-bit of the result. In this work, we use the second probability truncation technique. First, the parties generate a random truncation pair $\left(⟦s^{\prime }⟧,⟦s⟧\right)$ in the preprocessing phase, where $s^{\prime }\in {\left\{0,1\right\}}^{\ell }$ with 2f-bit precision, $s\in {\left\{0,1\right\}}^{f}$ with *f*-bit precision, such that $s=s^{\prime }/2^{f}$. In the online phase, the parties jointly compute $⟦z^{\prime }-s^{\prime }⟧$, then compute and open $\left(z^{\prime }-s^{\prime }\right)$, which is followed by computing $⟦z⟧=⟦s⟧+\left(z^{\prime }-s^{\prime }\right)/2^{f}$ locally. The truncation induces error is only $2^{-f}$.

### 3.3. Oblivious Selection Protocol

As an important part of our privacy-preserving *K*-means clustering protocol, we define the oblivious selection functionality $F_{\mathrm{OS}}$, whose functionality takes the arithmetic shares ⟦x⟧, ⟦y⟧ and boolean share $⟦b{⟧}^{B}$ as input, and returns ⟦x⟧ if b=0, and ⟦y⟧ otherwise. Note that $F_{\mathrm{OS}}$ can be explained as f(x,y,b)=(1−b)x+by=x+(y−x)b. It seems that the parties only need to compute multiplication between (y−x) and *b* once to implement $F_{\mathrm{OS}}$. However, this is non-trivial since $⟦y-x{⟧}^{A}$ is arithmetic share and $⟦b{⟧}^{B}$ is boolean share, while the RSS multiplication only works for same shares.

A natural idea is to convert $⟦b{⟧}^{B}$ to $⟦b{⟧}^{A}$ by using bit injection protocol $\prod_{B}2A$ from ABY3 [31], where three-party OT is required. Instead of using OT, we implement conversion with the Beaver trick [35]. Suppose that the parties access the precomputed conversion, and obtain precomputed random bit share $⟦c{⟧}^{B}$ and $⟦c{⟧}^{A}$ in the preprocessing phase. In the online phase, the parties compute and reconstruct the bit e=b⊕c, followed by setting $⟦d{⟧}^{A}=⟦1-c{⟧}^{A}$ if e=1, and $⟦d{⟧}^{A}=⟦c{⟧}^{A}$ otherwise. Finally, the parties compute $⟦z{⟧}^{A}=⟦\left(y-x\right)\cdot d{⟧}^{A}+⟦x{⟧}^{A}$, where $⟦\left(y-x\right)\cdot d{⟧}^{A}$ can be computed by using RSS multiplication $\prod_{\mathrm{Mul}}$ between $⟦y-x{⟧}^{A}$ and $⟦x{⟧}^{A}$.

Since that random bit $⟦c{⟧}^{B}$ and $⟦c{⟧}^{A}$ are used as the one-time pad, the corrupted party can not learn any information about *b*. Even though the parties reveal the masked value *e* in the clear, our oblivious selection protocol is still secure. Looking ahead, the oblivious selection protocol is used to find the index of minimum component from vector and generate one-hot vector.

### 3.4. Secure Euclidean Squared Distance Protocol

From Section 2.3, it is important to compute the Euclidean distance between two points using Equation (1). However, the square root is unfriendly to construct the SS-based protocol since it is a nonlinear operation and has an expensive communication overhead. Note that $f\left(x\right)=\sqrt{x}$ is a monotonically increasing function, which can be replaced with f(x)=x. This would, however, not affect the results on clustering since the only thing we need here is the relationship of size between two values. In this way, we replace Equation (1) with the following Euclidean squared distance equation:

(4) $$X_{ik}=\sum_{j=1}^{d}{\left(P_{ij}-\phi_{kj}\right)}^{2}.$$

Similarly, Equation (3) can be replaced with the following Equation (5):

(5) $$e=\sum_{k=1}^{K}e_{k}=\sum_{k=1}^{K}\sum_{j=1}^{d}{\left(\varphi_{kj}-\phi_{kj}\right)}^{2}.$$

Now, we focus on, given the share of vector $x=(x_{1},\cdots ,x_{d})$ and $y=(y_{1},\cdots ,y_{d})$, how to compute the share of Euclidean squared distance. We define this functionality as $F_{\mathrm{ESD}}$. Observe that this can be done by first computing $z=x-y=(x_{1}-y_{1},\cdots ,x_{d}-y_{d})$, and then computing the inner product between z and z. A naive way is for the parties to invoke RSS multiplication *d* times for *d*-dimension, consume *d* truncation pair to truncate the result, locally sum the result, and reshare. The communication is O(d) ring elements, which is dependent on *d*, the size of vector.

In this work, we use the delay re-share technique [31] to reduce communication complexity for inner product, which is only communication O(1) ring element and independent of *d*.

Let $\left(⟦s^{\prime }⟧,⟦s⟧\right)$ be the shares of truncation pair among the parties, where $s^{\prime }\in {\left\{0,1\right\}}^{\ell }$ with 2f-bit precision, $s\in {\left\{0,1\right\}}^{f}$ with *f*-bit precision, such that $s=s^{\prime }/2^{f}$. The delay re-share technique can be explained as the following Equation (6):

(6) $$⟦x⟧\cdot ⟦y⟧=\mathrm{reconstruct}\left(\left(\sum_{j=1}^{d}⟦x_{j}⟧\cdot ⟦y_{j}⟧\right)+⟦s^{\prime }⟧\right)/2^{f}-⟦s⟧.$$

In a word, the parties first compute a 3-out-of-3 additive sharing of each $⟦x_{i}⟧⟦y_{i}⟧$ locally, then sum together, mask, truncate, and reshare the final result for 2-out-of-3 replicated sharing semantics. It is easy to see that this would only require communicate 1 ring element per party, which is independent of *d*. Furthermore, the truncation-induced error is only $2^{-f}$ with respect to the overall inner product.

**Figure 1:**
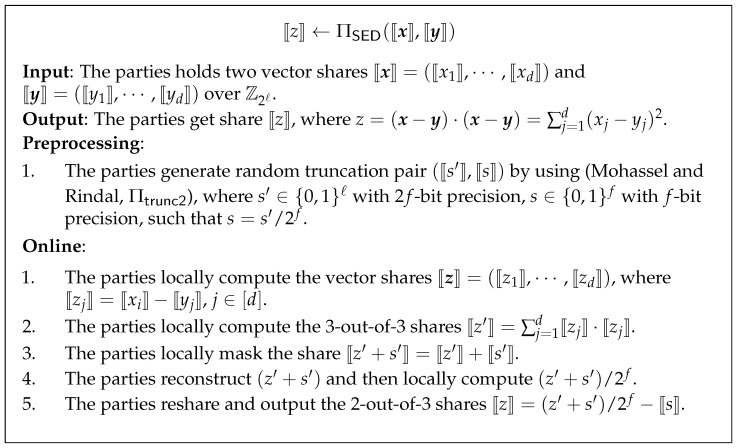
Secure Euclidean squared distance protocol [31].

The protocol for secure Euclidean squared distance is described in Figure 1.

### 3.5. Secure Comparison Protocol

In order to obtain the relationship between two given values, we have to consider how to implement comparison when the values are shared. We define secure comparison functionality $F_{\mathrm{LT}}$, which takes ⟦x⟧ and ⟦y⟧ as input, return boolean share $⟦b{⟧}^{B}$, where bit b=1 if x<y, and b=0 otherwise.

Let a=(x−y), then *z* can be computed by extracting the most significant bit (MSB) of *a*, i.e., b=MSB(a). Instead of using optimized parallel-prefix-adder-based bit extraction protocol from ABY3 [31], Wagh et al. [32] present a more efficient alternative method.

Recall that $⟦a⟧:=(a_{1},a_{2},a_{3})$, and $a\equiv a_{1}+a_{2}+a_{3}\;\mathrm{mod}\;2^{\ell }$, one has

(7) $$b=\mathrm{MSB}\left(a\right)=\mathrm{MSB}\left(a_{1}\right)⊕\mathrm{MSB}\left(a_{2}\right)⊕\mathrm{MSB}\left(a_{3}\right)⊕c,$$

where c∈{0,1} is a carry bit from (ℓ−2)-th index and can be computed by Equation (8).

(8) $$c=\left\{\begin{array}{cc} 1, & \mathrm{if}\;2^{\ell -1}\leq a_{1}+a_{2}+a_{3}<2^{\ell },\\ 0, & \mathrm{Otherwise}. \end{array}\right.$$

From Equation (7), we observe that $P_{i}$ can compute $\mathrm{MSB}(a_{i})$ locally; thus, the main challenge here is how to compute *c* in a secure way. Note that Equation (8) is also equivalent to $c=(\left(2a_{1}+2a_{2}+2a_{3}\right)\geq_{?}2^{\ell })$, which can be computed by wrap function. Wagh et al. [32] give us a solution for wrap function, denoted as $\prod_{\mathrm{WA}}$. We refer the reader to their work for correctness and security.

**Figure 2:**
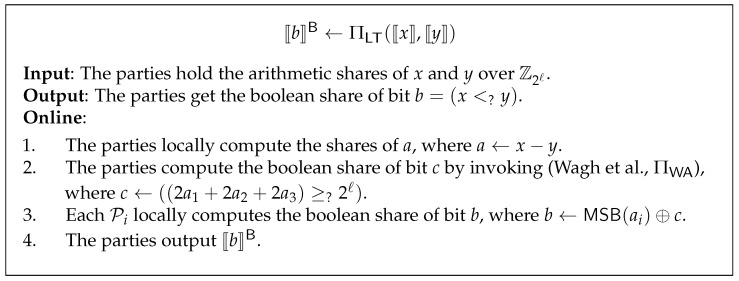
Secure comparison protocol [32].

The secure comparison protocol is described in Figure 2. Furthermore, if one of the secrets is known to all parties, e.g., *y*, the share of *y* can be defined by letting $⟦y⟧:=(y+\alpha_{1},\alpha_{2},\alpha_{3})$ without any interaction, where $\left(\alpha_{1},\alpha_{2},\alpha_{3}\right)$ is zero sharing generated by PRF keys among the parties. Thus, the secure comparison protocol is also work. We denote this case as $⟦b{⟧}^{B}\leftarrow F_{\mathrm{LT}}\left(⟦x⟧,y\right)$.

### 3.6. Secure Assignment Protocol

**Figure 3:**
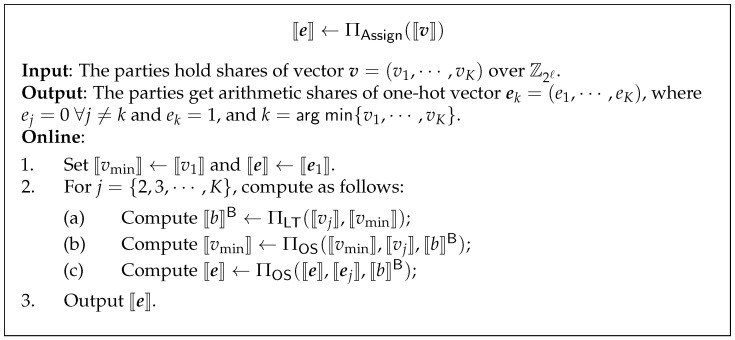
Secure Assignment protocol.

Recall that once the parties obtain the shares of the Euclidean squared distance between a point and all centorids, the following step is to assign this point to the closest cluster. This step can be abstracted as the question of, given *K*-dimension secret shared vector $⟦v⟧=(⟦v_{1}⟧,\cdots ,⟦v_{K}⟧)$, how to compute the secret shared one-hot vector e, where ‘1’ appears in the *k*-th component, and $k=\arg\;\min\{v_{1},\cdots ,v_{K}\}$. We denote secure assignment functionality as $F_{\mathrm{Assign}}$. The idea is straightforward. We implement secure assignment functionality with the following protocol $\prod_{\mathrm{Assign}}$ (see Figure 3).

### 3.7. Secure Division Protocol

As shown in Section 2.3, the parties need to recalculate the average vector of the points in each cluster in a secure way. Note that the average can be split by computing addition and division, where addition can be computed locally, hence the key point is to compute division. If the divisor is known to all parties, then division can be computed locally. However, observe that the divisor denotes the number of each cluster point and should be protected since full data privacy is required. Thus, the computation of division becomes difficult in our scenarios. We define secure division functionality $F_{\mathrm{Div}}$ as follows: Given secret shared value ⟦a⟧ and ⟦b⟧ with $b\in Z^{+}$, the parties compute the share ⟦c⟧, such that c=a/b.

Instead of invoking division garbled circuit protocol, we implement division with numerical method. The numerical method is one of the most commonly used techniques for constructing SS-based secure protocol due to its efficiency. In this work, we implement division using Goldschmidt’s algorithm [36], which approximates the desired operation as a series of multiplication.

Let $w_{0}$ be an initial approximation of 1/b, and $\epsilon_{0}:=1-b\cdot w_{0}$ be the relative error for the approximation $w_{0}$ such that $|\epsilon_{0}|<1$. The Goldschmidt algorithm iteratively computes the following Equation (9):

(9) $$c=\frac{a}{b}=a\cdot \frac{1}{b}\approx a\cdot w_{0}\left(1+\epsilon_{0}\right)\left(1+\epsilon_{0}^{2}\right)\cdots \left(1+\epsilon_{0}^{2^{t-1}}\right),$$

where *t* is the number of iterations. When t→+∞, one has *c* converges to a/b [36]. We set t=2, which is sufficient for a close approximation with our choice of fixed-point precision.

**Figure 4:**
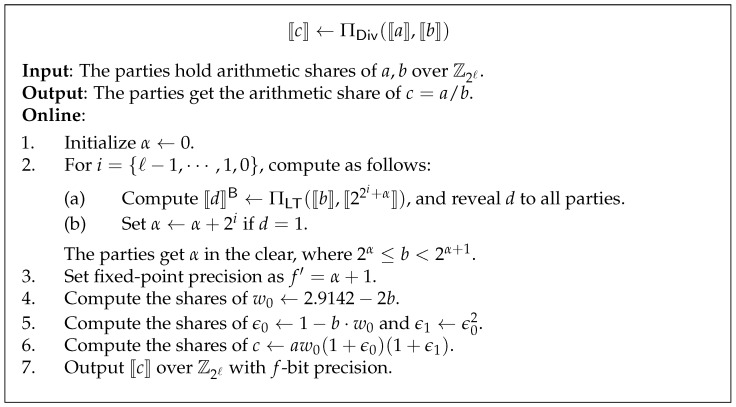
Secure division protocol [32].

Catrina and Saxena [34] give us a good initial approximation of $w_{0}$ in the interval [0.5,1), that is $w_{0}=2.9142-2b$. However, the major challenge of our work is that $b\in Z^{+}$ does not belong to the interval [0.5,1). In this work, we use the technique proposed by Wagh et al. [32]. The key insight here is that *b* is interpreted as a value with (α+1)-bit fixed-point precision but not *f*-bit precision, where α∈Z such that $2^{\alpha }\leq b<2^{\alpha +1}$. Thus, one should first extract α. The secure division protocol is described in Figure 4. From step 1 and step 2, we extract and reveal α to all parties, which only leaks the range of *b* and nothing else.

## 4. Privacy-Preserving K-Means Clustering

We now give a formal description of our privacy-preserving *K*-means clustering protocol, following the basic building blocks outlined above.

### 4.1. Secret Distribution

Recall that all data are held by the data owners in the secure outsourced scenarios, thus secret distribution phase is completed by the data owner. This implies that all data are *horizontal partitioned*. As an optimized, instead of generating 2-out-of-3 replicated shares directly, the data owner generates 3-out-of-3 additive shares and then sends to three computing parties/servers, who reshare the shares and obtain valid 2-out-of-3 replicated shares. In this way, we reduce communication costs of the data owner by a half.

### 4.2. Cluster Initialization

As shown in Section 2.3, we need to initialize *K* centroids before the Lloyd’s steps. In this work, we assume that the data owner chooses *K* random points as the initialized centroids and secret share to three computing parties for simple. In this way, cluster initialization can be combined with secret distribution.

### 4.3. Lloyd’s Steps

#### 4.3.1. Approximation of Euclidean Distance

Recall that we replace Euclidean distance with Euclidean squared distance, which is not affect the final result as we shown in Section 3.4. For i∈[n] and k∈[K], the parties first invoke $F_{\mathrm{SED}}$ to compute the shares of Euclidean squared distance $X_{ik}$ between data point $P_{i}$ and centroid $\phi_{k}$, and then form n×K matrix X.

#### 4.3.2. Assigning Data Points to the Closest Cluster

For i∈[n], we denote $X_{i}$ as the *i*-th row vector of X. In order to assign data point $P_{i}$ to the closet cluster, the parties invoke our $F_{\mathrm{Assign}}$ protocol as described in Section 3.6. The one-hot vector output $⟦c_{i}⟧\leftarrow F_{\mathrm{Assign}}\left(⟦X_{i}⟧\right)$ indicates which cluster center this data point is assigned to. We form K×n cluster matrix ⟦C⟧, such that the *i*-th column of C is $c_{i}$.

#### 4.3.3. Recalculating Cluster Centers

Given cluster matrix C, the parties need to recalculate each centroid. Observe that for each k∈[K], the *k*-th cluster center has $D_{k}=\sum_{i=1}^{n}C_{ki}$ points exactly. Instead of computing $\varphi_{k}=\frac{C_{k}\cdot P}{D_{k}}$ separately, one can first compute M=C·P, and then compute $\varphi_{k}=\frac{M_{k}}{D_{k}}$, where $M_{k}$ is the *k*-th row of M. Given secret shared matrix ⟦C⟧, the new centroid matrix φ can be computed by vectorized multiplication technique [31] and secure division protocol (as described in Section 3.7). Furthermore, the parties also compute the shares of Euclidean squared distance $e_{k}$ between $\varphi_{k}$ and $\phi_{k}$ for checking the stopping criterion.

#### 4.3.4. Checking the Stopping Criterion and Updating Centroids

In order to check the stopping criterion, the parties first locally compute $e=\sum_{k=1}^{K}e_{k}$, and then compare with a given small error ε, stop the criterion if e<ε, otherwise update φ and continue next round. This can be done by invoking secure comparison protocol $\prod_{\mathrm{LT}}$. The only reveal message is $b=(e{<}_{?}\varepsilon )$, which does not affect full data privacy.

### 4.4. Main Construction

**Figure 5:**
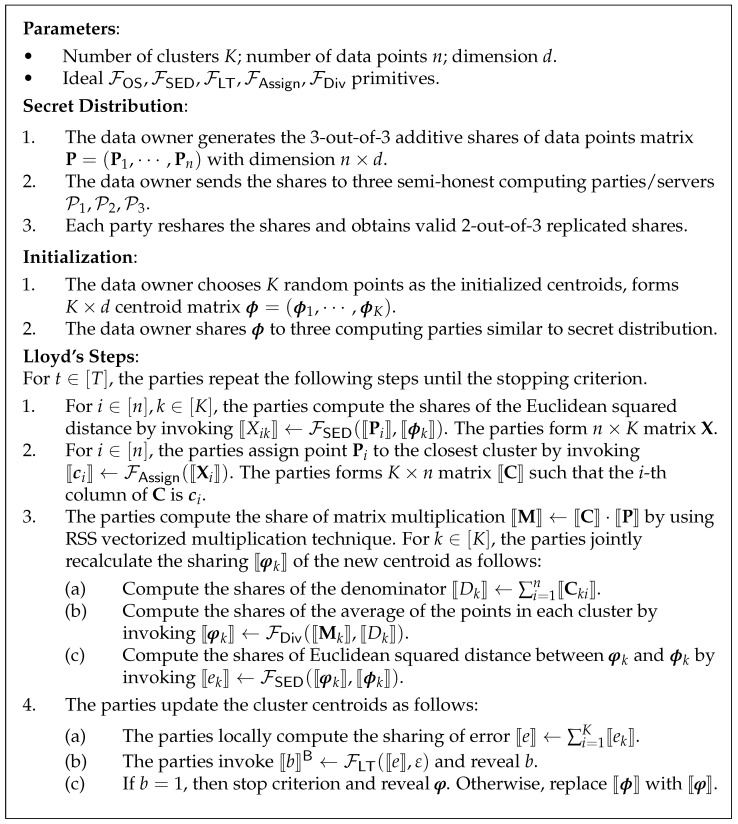
Our Privacy-preserving *K*-means Protocol.

The secure *K*-means protocol is described in Figure 5. According to the definition of full data privacy, the information about the intermediate centroids, cluster assignments, and cluster sizes should be protected. From Figure 5, we can see that the only information we leak is the range of $D_{k}$ and nothing else. Therefore, our construction provides full data privacy.

## 5. Security Analyses

Our protocol follows the universally composable framework [29] and provides security against a single corrupted party under the semi-honest model. The universally composable framework guarantees the security of arbitrary composition of different protocols. Therefore, we only need to prove the security of individual protocols.

**Theorem** **1.** *$\prod_{\mathrm{OS}},\prod_{\mathrm{SED}},\prod_{\mathrm{LT}},\prod_{\mathrm{Assign}},\prod_{\mathrm{Div}}$ securely realizes $F_{\mathrm{OS}},F_{\mathrm{SED}},F_{\mathrm{LT}},F_{\mathrm{Assign}},F_{\mathrm{Div}}$, respectively, in the presence of one semi-honest corrupt party under the hybrid model.*

**Proof of Theorem** **1.** We list the security of those protocols as follows:

*Security for $\prod_{\mathrm{OS}}$*: This can be reduced to the security of one-time pad and RSS multiplication $\prod_{\mathrm{Mul}}$.

*Security for $\prod_{\mathrm{SED}}$*: This protocol is based on the vectorized multiplication protocol [31], which has been proven secure under the semi-honest model.

*Security for $\prod_{\mathrm{LT}}$*: This can be reduced to the security of wrap function protocol, which has been proven secure in [32].

*Security for $\prod_{\mathrm{Assign}}$*: This can be reduced to the security of $\prod_{\mathrm{LT}}$ and $\prod_{\mathrm{OS}}$.

*Security for $\prod_{\mathrm{Div}}$*: This protocol has been proven secure in [32]. □

**Theorem** **2.** *The protocol in Figure 5 securely computes the K-means clustering under the semi-honest model, given the ideal $F_{\mathrm{SED}},F_{\mathrm{Assign}},F_{\mathrm{Mul}},F_{\mathrm{LT}}$, and $F_{\mathrm{Div}}$ functionalities, respectively.*

**Proof of Theorem** **2.** We exhibit a simulator Sim for simulating a corrupt party $P_{1}$. The simulator for $P_{2}$ and $P_{3}$ should be the same as $P_{1}$.

Sim simulates the view of corrupt $P_{1}$, which consists of his input/output and received messages, and proceeds as follows:

1. Calls $F_{\mathrm{SED}}$ simulator ${\mathrm{Sim}}_{F_{\mathrm{SED}}}\left(⟦P_{i}⟧,⟦\phi_{k}⟧\right)$ to simulate step 1, then appends its output to the view;
2. Calls $F_{\mathrm{Assign}}$ simulator ${\mathrm{Sim}}_{F_{\mathrm{Assign}}}(⟦X_{i}⟧$ to simulate step 2, then appends its output to the view;
3. Calls $F_{\mathrm{Mul}}$ simulator ${\mathrm{Sim}}_{F_{\mathrm{Mul}}}\left(⟦C⟧,⟦P⟧\right)$ to simulate step 3, then appends its output to the view;
4. Calls $F_{\mathrm{Div}}$ simulator ${\mathrm{Sim}}_{F_{\mathrm{Div}}}\left(⟦M_{k}⟧,⟦D_{k}⟧\right)$ to simulate step 3(b), then appends its output to the view.
5. Calls $F_{\mathrm{LT}}$ simulator ${\mathrm{Sim}}_{F_{\mathrm{LT}}}\left(⟦e⟧,\varepsilon \right)$ to simulate step 4(b), then appends its output to the view.

We argue the indistinguishability of the produced transcript from the execution of real world. At first, we formally show the simulation by proceeding the sequence of hybrid transcripts $T_{0}$, $T_{1}$, $T_{2}$, $T_{3}$, $T_{4}$, $T_{5}$, where $T_{0}$ is the real view of A, and $T_{5}$ is the output of Sim.

*Hybrid 1.* Let $T_{1}$ be the same as $T_{0}$, except the $F_{\mathrm{SED}}$ execution is replaced with running the simulator ${\mathrm{Sim}}_{F_{\mathrm{SED}}}\left(⟦P_{i}⟧,⟦\phi_{k}⟧\right)$. Because $\prod_{\mathrm{SED}}$ has been proven secure, thus ${\mathrm{Sim}}_{F_{\mathrm{SED}}}$ is guaranteed to produce output indistinguishable from the execution of real world. Therefore, $T_{1}$ and $T_{0}$ are indistinguishable.

*Hybrid 2.* Let $T_{2}$ be the same as $T_{1}$, except the $F_{\mathrm{Assign}}$ execution is replaced with running the simulator ${\mathrm{Sim}}_{F_{\mathrm{Assign}}}\left(⟦X_{i}⟧\right)$. This functionality outputs the share of the Euclidean squared distance between $P_{i}$ and $\phi_{k}$, which does not reveal any information about the result. Moreover, the output of ${\mathrm{Sim}}_{F_{\mathrm{Assign}}}$ is indistinguishable from the execution of real world, thus $T_{2}$ and $T_{1}$ are indistinguishable.

*Hybrid 3.* Let $T_{3}$ be the same as $T_{2}$, except the $F_{\mathrm{Mul}}$ execution is replaced with running the simulator ${\mathrm{Sim}}_{F_{\mathrm{Mul}}}\left(⟦C⟧,⟦P⟧\right)$. Because $\prod_{\mathrm{Mul}}$ has been proven secure, ${\mathrm{Sim}}_{F_{\mathrm{Mul}}}$ is guaranteed to produce output indistinguishable from the execution of the real world. Therefore, $T_{3}$ and $T_{2}$ are indistinguishable.

*Hybrid 4.* Let $T_{4}$ be the same as $T_{3}$, except the $F_{\mathrm{Div}}$ execution is replaced with running the simulator ${\mathrm{Sim}}_{F_{\mathrm{Div}}}\left(⟦M_{k}⟧,⟦D_{k}⟧\right)$. This is because $\prod_{\mathrm{Div}}$ has been proven secure, and its output is the share, which is indistinguishable from the pseudo-randomness. In other words, the output of ${\mathrm{Sim}}_{F_{\mathrm{Div}}}$ is indistinguishable from the execution of real world. Thus, $T_{4}$ and $T_{3}$ are indistinguishable.

*Hybrid 5.* Let $T_{5}$ be the same as $T_{4}$, except the $F_{\mathrm{LT}}$ execution is replaced with running the simulator ${\mathrm{Sim}}_{F_{\mathrm{LT}}}\left(⟦e⟧,\varepsilon \right)$. The output is the share, which does not reveal any information about the data points. In addition, the output of ${\mathrm{Sim}}_{F_{\mathrm{LT}}}$ is indistinguishable from the execution of real world. Thus $T_{5}$ and $T_{4}$ are indistinguishable.

In summary, $T_{0}$ and $T_{5}$ are indistinguishable, which is end of the proof. □

## 6. Experiments

### 6.1. Experimental Setup

We implement our privacy-preserving clustering protocol and report the experimental results in this section. All experiments are executed on Ubuntu 22.04 LTS with Intel(R) Xeon(R) Gold 5222 CPU @3.41GHz and 256 GB RAM. All parties run in the same network and the connection is simulated using the Linux tc command. The LAN setting has 0.2 ms round-trip latency and 5 Gbps network bandwidth, while the WAN setting has 20 ms round-trip latency and 400 Mbps network bandwidth. We implement our protocol with the C++ open source framework FALCON (https://github.com/snwagh/falcon-public (accessed on 18 June 2022)) [32].

In all experiments, we assume the orginal data has been normalized in the same level. For the public parameters, we set bit-length ℓ=64, fixed-point precision f=13, and the number of iteration t=2. Table 2 summarizes the real datasets used in our experiments. Furthermore, we also compare with Mohassel et al. [27] in the self-generated dataset.

### 6.2. Accuracy

In order to evaluate the accuracy of clustering classification for the real dataset, we usually compare it to the ground truth model. We downloaded the ground truth model of Iris and arff from Github repository (https://github.com/deric/clustering-benchmark (accessed on 18 June 2022)). Note that the standard *K*-means clustering is sensitive to the initialized centroids; thus, we ran the algorithm many times with different initialized centroids and take the best result as the global optimal solution.

**Figure 6:**
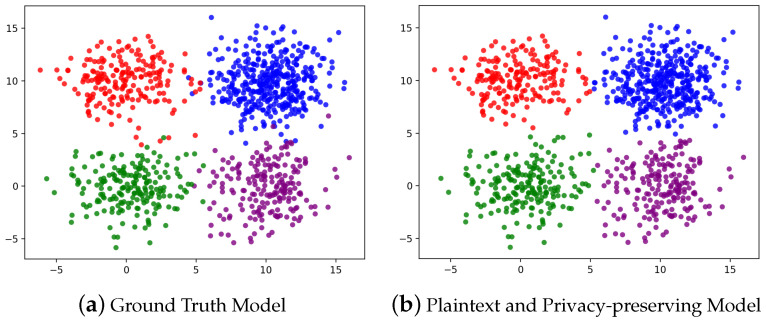
Comparison of accuracy for ground truth, plaintext, and privacy-preserving model for 2D dataset arff. Our privacy-preserving model reaches the same accuracy as the plaintext model. The accuracy is 98.20% compared to the ground truth model.

We used 2D dataset arff and 4D dataset Iris for evaluating accuracy and report the experimental results in Table 2. For a visual comparison, the experimental result of dataset arff is shown in Figure 6. Compared to the ground truth model of dataset arff, we reached 98.20% accuracy in our privacy-preserving model. For dataset Iris, we reached 92.67% accuracy in our privacy-preserving model. Both accuracy results are the same as the plaintext algorithm; hence, our privacy-preserving protocol is feasible.

Recall that our secure division protocol adopts the numerical method and the result of the secure multiplication protocol needs to be truncated; thus, the privacy-preserving result φ is only approximate to the plain result but not the exact result. In fact, our experiment shows that the relative error is about $10^{-2}$, which means this part has a negligible impact on model accuracy compare with the plaintext algorithm. In other words, our privacy-preserving protocol is feasible, even if φ is only approximate to the truth value. For the large-scale dataset, we argue that the relative error can be improved by taking bigger public parameters *ℓ*, *f*, and *t*. However, it will require more runtime and communication cost. There is a trade-off between runtime and accuracy of the division. We do not consider the accuracy of experiment in the following section.

### 6.3. Runtime and Communication

In this section, we focus on the total communication cost and runtime of our privacy-preserving protocol. We ran each experiment five times and computed the average of wall clock runtime as the reported runtime. We report the experimental results in Table 3. Note that iteration *T* depends on the initialized clustering centroids; hence, we set it to a fixed value in this experiment (say, T=10).

As shown in Table 3, the runtime and communication overhead are independent from the dimension *d*; this is because we enjoy the benefit from the vectorized multiplication and delay re-share technique [31].

Even though n=100,000, our scheme lasted less than 8 min and communicated less than 400 MB in total under the LAN setting. Although the overall communication cost is low, we observe that the runtime of our scheme is not good at the WAN setting. The reason for this is because WAN has low-bandwidth and high-latency, while the secret-sharing-based schemes usually have much communication rounds that is bad for this setting. Thus, we argue that our privacy-preserving scheme is practical when the network is fast, high-bandwidth, and low-latency. We estimate that our privacy-preserving protocol can be used to deal with datasets of million points in an acceptable time (for example, within 2 h for clustering one million points to 2 groups in the LAN setting).

### 6.4. Comparison with Mohassel et al. [27]

Recall that Mohassel et al. [27] also provides full data privacy guarantees (see Table 1); thus, we also compare to their work with the self-generated dataset in our experiment environment. We downloaded the code of Mohassel et al. [27] from their Github repository (https://github.com/osu-crypto/secure-kmean-clustering (accessed on 18 June 2022)). In order to save time, all experiments are only considered under the localhost setting, which has 0.027ms round-trip latency and 41.1Gbps network bandwidth. We ran each protocol five times and report the average of wall clock runtime and communication costs. Instead of running the code of Mohassel et al. [27] in every iteration, we measured its runtime for one round iteration and multiplied by the number of iterations *T* to save time.

Table 4 presents the computation cost and communication cost of our protocol compared with [27]. Our experimental results demonstrate that the computation costs of our protocol is about 16.5×–25.2× faster than [27], and the communication cost is about 63.8×–68.0× less than [27]. This is because their construction relies heavily on garbled circuit and oblivious transfer, while our scheme is only based on replicated secret sharing.

## 7. Conclusions and Future Work

In this work, we presented an efficient RSS-based privacy-preserving *K*-means clustering scheme over $Z_{2^{\ell }}$ under the semi-honest model. Our scheme provides full data privacy. The experiment report shows that our protocol is highly efficient and practical, as well as suitable for large-scale clustering tasks when the network is fast. Therefore, we argue that our scheme is suitable for secret-sharing-based secure outsourced computation.

The next direction of future work can extend our scheme to the malicious adversarial setting, which will be a non-trivial problem. This is because malicious adversary may not follow protocol specifying and deviate arbitrarily in any phase. For example, the adversary makes the corrupted party sends incorrect messages, such that it can break the correctness of protocol. Therefore, we should ensure that the sending message of the parties are correct. A promising direction for this case is to introduce the SPDZ protocol [37], where the correctness of the sending message can be protected by using message authentication codes (MACs).

## Figures and Tables

**Table 1:** The comparison of different SS-based *K*-means schemes. ASS: Additive secret sharing, CRT-SS: Chinese remainder theorem secret sharing, SSS: Shamir’s secret sharing, RSS: Replicated secret sharing. ZKP: Zero knowledge proof, GC: Garbled circuit. OT: Oblivious transfer. N/A: Undefined. L1: intermediate centroids, L2: intermediate cluster sizes, L3: other intermediate values (e.g., intermediate cluster assignments or distance comparison results). ✓: no leakage, ✗: leakage. FDP: Full data privacy.

Scheme	Security	Technology	Domain	L1	L2	L3	FDP
Doganay et al. [22]	Semi-honest	ASS	N/A	✓	✓	✗	✗
Upmanyu et al. [25]	Semi-honest	CRT-SS	$Z_{p}$	✓	✗	✗	✗
Patel et al. [23]	Semi-honest	SSS	$Z_{p}$	✗	✗	✓	✗
Patel et al. [24]	Malicious	SSS+ZKP	$Z_{p}$	✗	✗	✓	✗
Baby and Chandra [26]	N/A	CRT-SS	$Z_{p}$	✗	✗	✗	✗
Mohassel et al. [27]	Semi-honest	ASS+GC+OT	$Z_{2^{\ell }}$	✓	✓	✓	✓
**This work**	Semi-honest	RSS	$Z_{2^{\ell }}$	✓	✓	✓	✓

**Table 2:** Descriptions of the datasets we used in experiments, where *n* is the number of data points, *K* is the number of clusters, and *d* is the dimension. We also report the accuracy of different datasets, if the ground truth model of dataset exists.

Dataset	*n*	*K*	*d*	Accuracy
Iris	150	3	4	92.67%
arff	1000	4	2	98.20%
Self-generated	{10,000,100,000}	{2,5}	{5,10,15,20}	—

**Table 3:** The comparison of wall clock runtime and communication cost with different dimensions in the self-generated dataset both with LAN and WAN setting, where *n* is the number of data points, *K* is the number of clusters, *d* is the dimension, and the iteration T=10.

Parameters			Runtime		Comm. (MB)
*n*	*K*	*d*	LAN (s)	WAN (min)	
10,000	2	5	63.8160	134.3737	37.1516
		10	63.8020	134.3832	37.2976
		20	63.6132	134.3962	37.5896
	5	5	160.9406	336.0150	134.2790
		10	161.2164	336.1254	134.6440
		20	161.3586	336.2652	135.3740
100,000	2	5	474.7150	1336.1333	370.1520
		10	473.9687	1336.1968	370.2980
		20	475.1037	1336.2415	370.5900

**Table 4:** Comparison with Mohassel et al. [27] in large-scale self-generated datasets under the localhost setting, where *n* is the number of data points, *K* is the number of clusters, and dimension d=2.

Parameters			Runtime (min)			Communication (MB)		
*n*	*K*	*T*	[27]	This Work	Improved Factor	[27]	This Work	Improved Factor
$10^{4}$	2	10	1.77	**0.09**	19.5×	2377	**37**	64.1×
		20	3.36	**0.19**	17.9×	4733	**74**	63.8×
	5	10	4.69	**0.28**	16.5×	9121	**134**	68.0×
		20	9.46	**0.56**	16.9×	18220	**268**	68.0×
$10^{5}$	2	10	15.33	**0.74**	20.8×	23731	**370**	64.1×
		20	29.74	**1.15**	20.4×	47262	**740**	63.9×
	5	10	46.51	**1.85**	25.2×	91128	**1339**	68.0×
		20	91.61	**3.65**	25.1×	181867	**2678**	67.9×

## Data Availability

Not applicable.
